# A Novel Mitochondrial Targeted Compound Phosundoxin Showing Potent Antifungal Activity against Common Clinical Pathogenic Fungi

**DOI:** 10.3390/jof10010028

**Published:** 2023-12-31

**Authors:** Shu Zhang, Yuanyuan Geng, Bin Wei, Yangzhen Lu, Lihua He, Fei Zhao, Jianzhong Zhang, Zhaohai Qin, Jie Gong

**Affiliations:** 1 National Key Laboratory of Intelligent Tracking and Forecasting for Infectious Diseases, National Institute for Communicable Disease Control and Prevention, Chinese Center for Disease Control and Prevention, Beijing 102206, China; zhangshu@icdc.cn (S.Z.);; 2 National Institute for Communicable Disease Control and Prevention Joint Laboratory of Pathogenic Fungi, Peking University First Hospital, Beijing 102206, China; 3 College of Science, China Agricultural University, Beijing 100193, China; 4 Collaborative Innovation Center of Recovery and Reconstruction of Degraded Ecosystem in Wanjiang Basin Co-Founded by Anhui Province and Ministry of Education, School of Ecology and Environment, Anhui Normal University, Wuhu 241002, China

**Keywords:** fungi, antifungal activity, transcriptome, mechanisms, novel compound

## Abstract

The current increase in resistance to antifungal drugs indicates that there is an urgent need to explore novel antifungal drugs with different mechanisms of action. Phosundoxin is a biphenyl aliphatic amide using a TPP-targeting strategy which targets mitochondria. To provide insights into the antifungal activities of phosundoxin, the antifungal susceptibility testing of phosundoxin was conducted on 158 pathogenic fungi and compared to that of traditional azole drugs. Phosundoxin displayed a broad-spectrum antifungal activity on all the tested yeast-like and filamentous fungi ranging from 2 to 16 mg/L. In particular, azole-resistant clinical isolates of *Candida albicans* were susceptible to phosundoxin with the same MICs as azole-susceptible *C. albicans*. Transcriptome analysis on azole-resistant *C. albicans* identified 554 DEGs after treatment with phosundoxin. By integrating GO and KEGG pathway enrichment analysis, the antifungal activity of phosundoxin was related to impairment of mitochondrial respiratory chain function. Acute oral and percutaneous toxicity of phosundoxin to rats showed that the compound phosundoxin were mild toxicity and LD_50_ was above 5000 mg/kg body weight in rats. This study demonstrated the potential of phosundoxin as an antifungal agent for the treatment of common fungal infection and contributed to providing insights into the mechanisms of action of phosundoxin against *C. albicans*.

## 1. Introduction

Pathogenic fungi have become relevant threats to human security [1,2]. A significant number of threats are derived from drug resistance or even multiple drug resistance of pathogenic fungi [3]. Azole drugs, for example, have resulted in a variety of fluconazole- and voriconazole-resistant *Candida* and *Aspergillus* formed through ergosterol biosynthetic enzyme ERG11 (Cyp51A) substitutions [4,5]. Therefore, it is important to explore novel, specific, and less-toxic drugs using new molecular targets in the treatment of fungal infections [6].

Mitochondria are suitable potential targets as they are present in most fungal cells. In addition to being responsible for upwards of 90% of cellular ATP production, these organelles play several roles, including the generation and regulation of reactive oxygen species, programmed cell death, and metabolic processes (including those related to amino acids, lipids, and nucleotides) [7]. Based on these roles, many mitochondrially targeted antifungal drugs have been developed [8,9].

**Figure 1:**
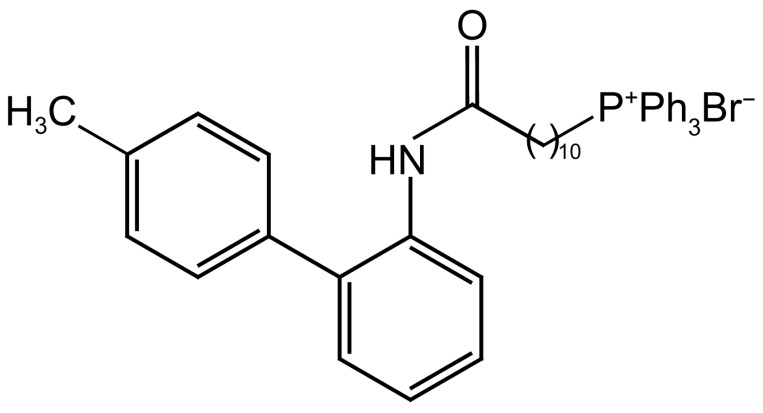
Chemical structure of phosundoxin.

Triphenylphosphonium cation (TPP^+^) is a lipophilic cation. It passes easily through phospholipid bilayers due to high lipophilicity and stable cationic charge, and potential gradient drives the accumulation of TPP^+^-conjugate in the mitochondrial matrix [10]. Thus, TPP-based mitochondrially targeted drugs have been designed, such as antioxidants and anticancer drugs leveraging these characteristics [11,12,13]. Previously, Wang et al. synthesized a series of antifungal boscalid analogues using a TPP-targeting strategy [14]. In particular, (11-((4′-methyl-[1,1′-biphenyl]-2-yl)amino)-11-oxoundecyl)-triphenylphosphonium bromide, a novel compound based on TPP^+^ and boscalid analogues, named phosundoxin (Figure 1), was found to be an effective inhibitor of the growth of common fungal pathogens in previous experimental studies [15]. However, research into its antifungal mechanism remained incomplete. In this study, the antifungal activity and transcriptome of phosundoxin were investigated to explore its efficacy and prospects as a novel antifungal drug.

## 2. Materials and Methods

### 2.1. Fungal Organisms

A total of 158 strains were involved in this study (Table 1), including 130 strains of *Candida* spp., 10 strains of *Cryptococcus* spp., 4 strains of *Aspergillus* spp., 6 strains of *Trichophyton* spp., and 8 strains of *Talaromyces marneffei*. The fungal isolates were stored at −80 °C at the Department of Fungal Diseases from National Institute for Communicable Disease Control and Prevention, Chinese Center for Disease Control and Prevention.

### 2.2. Antifungal Susceptibility Testing

Phosundoxin stock solution was obtained from China Agricultural University and was prepared in DMSO. The range of phosundoxin concentrations tested was 0.25–32 mg/L. Minimum inhibitory concentrations (MICs) of the phosundoxin were determined according to a broth microdilution method based on the Clinical and Laboratory Standards Institute (CLSI) reference documents M27 [16] and M38 [17] for yeasts and filamentous fungi, respectively. The MIC of phosundoxin was defined as a well with a 100% decrease in growth compared to the drug-free control well. Susceptibility to fluconazole, itraconazole, and voriconazole was determined using the Sensititre^®^ YeastOne^®^ YO10 system (Trek Diagnostics, Cleveland, OH, USA). Geometric mean (GM) MICs, MIC_90_, and MIC_50_ values were ascertained for species where at least 8 strains of a species were tested.

### 2.3. Growth Speed and Spore Morphology

Fresh *C. albicans* isolates were used for inoculation in Sabouraud Dextrose Broth (SDB) medium in triplicate at 28 °C and shaking at 180 rpm. Cultures were sampled to determine the optical density measurements (OD) at a wavelength of 600 nm of *C. albicans* at different times (0, 6, 12, 24, 28, 36, 48, 60, 72, and 84 h). The influence of phosundoxin on the growth of *C. albicans* was determined by adding phosundoxin at a final concentration of 4 mg/L during the logarithmic growth phase (20 h).

### 2.4. RNA Extraction

*C. albicans* CDCF157 recovered in SDB at 28 °C. When OD_600_ reached 0.1, 1‰ of the fresh *C. albicans* cells were inoculated in 50 mL SDB in triplicate at 28 °C with shaking at 180 rpm for 22 h. Cells were treated with phosundoxin at a final concentration of 4 mg/L and incubated for 2 h. The cells treated with DMSO under the same conditions served as controls. Cells were collected via centrifugation at 8000 rpm and used for subsequent RNA extraction. Total RNA was extracted using TRIzol reagent (Cwbio, Taizhou, Jiangsu, China) as well as other regents including chloroform, isopropyl alcohol, and ethanol (Sinopharm, Shanghai, China) via the TRIzol method [18]. RNA degradation was monitored at 1% agarose gels. The concentration and purity of total RNA was determined by measuring UV absorbance with a NanoDrop 1000 Spectrophotometer (Thermo Fisher, Wilmington, DE, USA).

### 2.5. Transcriptome Sequencing and Analysis

Six samples with three biological replicates of *C. albicans* CDCF157 were prepared for cDNA library construction, transcriptome sequencing, and analysis conducted by Majorbio Biotech, Shanghai, China. The transcriptome library was prepared following steps in a Truseq^TM^ RNA sample preparation kit (llumina, San Diego, CA, USA). After constructing library, a paired-end RNA-seq sequencing library was sequenced with an Illumina NovaSeq 6000 sequencer (2 × 150 bp read length). The raw paired end reads were trimmed and quality controlled through use of fastp software (version 0.19.5) [19] with default parameters. Clean reads were then separately mapped to the *C. albicans* SC5314_A22 genome using Hisat2 software (version 2.1.0) [20].

During the identification of differentially expressed genes (DEGs), an absolute value of log_2_FoldChange ≥1 and false discovery rate (FDR) < 0.05 were used as the screening criteria on the basis of DEseq2 software (version 1.24.0) [21]. DEGs were subjected to gene ontology (GO) functional analysis and Kyoto encyclopedia of genes and genomes (KEGG) pathway analysis. GO enrichment and KEGG analysis were performed by Goatools software (version 0.6.5) and KOBAS software (version 2.1.1) [22].

### 2.6. qRT-PCR Analysis of Gene Expression

For qRT-PCR, cDNA was synthesized using 1 μg of RNA as a template and purified using a cDNA reverse transcription kit (Takara, Tokyo, Japan) according to the manufacturer’s instructions. The concentration and purity of cDNA was determined by measuring UV absorbance using a NanoDrop 1000 Spectrophotometer. qRT-PCR was performed using Evagreen (Biotium, Fremont, CA, USA) and qPCR Master Mix (Vazyme, Nanjing, Jiangsu, China) on an ABI QuantStudio 6 flex instrument (Thermo Fisher, USA). The primers used to detect gene expression levels were listed in Table S6. Target genes included 21 DEGs associated with oxidative phosphorylation (OXPHOS), mitochondria, replication, translation, and cell growth as well as 12 randomly selected DEGs. The actin gene was used as an internal reference gene. Error bars were used to indicate standard deviation. The concentration of the cDNA template was 20 ng/μL. Relative transcription levels of target genes were calculated using the relative quantitation (2^−ΔΔCT^) method [23]. Three biological replications and technical replications were performed.

### 2.7. Acute Toxicity of Phosundoxin to SD Rats

Ten Sprague Dawley (SD) rats (half male and half female, 8 to 10 weeks of age) purchased from Vital River Laboratory Animal Technology (Foshan, Guangdong, China, SCXK (Yue) 2020-0063) and ten SD rats, as above, purchased from BesTest Bio-Tech (Zhuhai, Guangdong, China, SCXK (Yue) 2020-0051) were used for acute oral and percutaneous toxicity tests, respectively.

The rats were kept in a colony cage at 20–26 °C with 40–70% relative humidity and 12 h of light and dark cycle and had free access to standard water and food (SCXK (Yue) 2021-0166). The experimental protocol was approved and performed by the Institutional Animal Care and Use Committee of Zhongke Yinghai Technology, Co., Ltd. (Foshan, Guangdong, China) and followed the rules of the Declaration of Helsinki (protocol code 20230516-rat-1, 20230516-rat-3).

Prior to the formal acute toxicity test, we performed a preliminary test and found that phosundoxin was administered to SD rats at a dose of 5000 mg/kg body weight of SD rats without animal death. Therefore, in the formal acute toxicity test, a specified dose of 5000 mg/kg body weight of SD rats was administered orally and percutaneously. In acute percutaneous toxicity test, phosundoxin was applied to the labeled area for about 24 h, after which the drug was removed with pure water.

The observation period of the acute toxicity test was 14 days. The first day of administration was set as D1. The animals were observed for a further 14 days (D2–D15). Clinical symptoms, death, and weight changes of each animal were observed and recorded. The time point of the early observation was 30 min, 4 h, 1 d, so that the occurrence time of toxic reactions could be observed. After that, these animals were maintained for a further 14 days with the observation above made daily (D2–D15). The median lethal dose (LD_50_) was calculated based on the death and mortality of the animals. The animals were weighed once on D1, D8, and D15. Gross dissection was performed on the animals that were alive at the end (D15).

## 3. Results

### 3.1. Antifungal Activity of Phosundoxin

We evaluated the antifungal susceptibilities of phosundoxin through determining its MIC against a series of strains. As shown in Table 1, phosundoxin demonstrated a broad-spectrum antifungal activity against several yeast-like fungi (*Candida* spp., *Cryptococcus* spp.), filamentous fungi (*Aspergillus* spp., *Trichophyton* spp.), and dimorphic fungi (*Talaromyces marneffei*), with MICs ranging from 2 to 16 mg/L.

MICs of phosundoxin against yeast-like fungi ranged from 2 to 16 mg/L. The growth of most yeasts was inhibited when treated with 2–4 mg/L of phosundoxin. For example, *C. albicans*, *C. tropicalis*, *C. krusei*, *C. parapsilosis*, *C. haemulonii*, etc., had MIC_90_ of 4 mg/L. However, a few *Candida* species, including *C. glabrata*, *C. nivariensis*, and *C. bracarensis*, had lower susceptibility to phosundoxin, with MICs ranging from 8 to 16 mg/L (Table 1). In filamentous fungi, phosundoxin had a lower activity, with MICs of 4 to 16 mg/L for *Trichophyton* and *Aspergillus*, and the MICs of *Aspergillus* (16 mg/L) were higher than dermatophytes (4–8 mg/L). Dimorphic fungi are a unique group of fungi that respond to shifts in temperature by converting between hyphae and yeast. They are observed as hyphae forms in the environment temperature (25–30 °C) and transformed into yeast-like forms with pathogenic ability at body temperature (35 °C–37 °C). *Talaromyces marneffei* is a typical dimorphic fungus, MIC_90_ of 4 mg/L in its hyphal phase and MIC_90_ of 8 mg/L in its yeast phase, indicating that the phosundoxin was more effective in inhibiting the hyphal phase of *Talaromyces marneffei* (Table 1).

The 18 strains *C. albicans* in Table 1 could be divided into 10 azole-susceptible strains and 8 azole-nonsusceptible strains according to their antifungal susceptibilities to azoles (Table 2). In the case of fluconazole, the GM MICs of susceptible strains were 0.73 mg/L, while the GM MICs of nonsusceptible strains were 16 mg/L. However, it was found that the MICs of phosundoxin for azole-susceptible and azole-nonsusceptible *C*. *albicans* were 4 mg/L (Table 2). This indicated that the antifungal mechanism of phosundoxin was different from azoles.

### 3.2. A Preliminary Analysis of Transcriptome Sequencing Data

To understand the molecular changes underlying the impact of phosundoxin more completely, transcriptome profiling assays were performed on the azole-resistant *C. albicans*, CDCF157. Samples under exposure to 4 mg/L phosundoxin comprised the experimental group (T_157). Samples not treated with phosundoxin were used as a control group (CK_157). The sequencing of six cDNA libraries (three of each group) generated 44.12 Gb of clean bases, and the average clean data obtained were over 6 Gb for each sample. Moreover, clean base Q20 and Q30 percentages were over 97% and 93%, respectively. The mapping ratios with the reference *C. albicans* SC5314 genome were >94% (Tables S1 and S2). Therefore, the sequencing data were high quality and suitable to further biological analysis.

**Figure 2:**
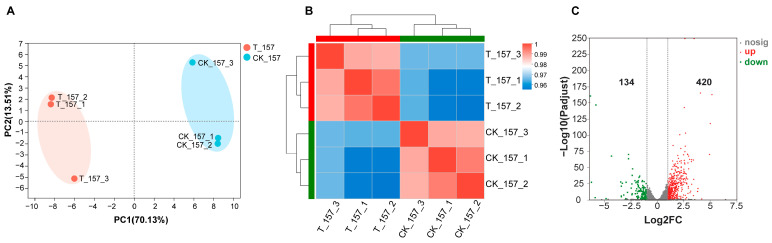
Validation of *C. albicans* transcriptome. (**A**) Principal component analysis (PCA) plot illustrating the level of correlation among T_157 (red) and CK_157 (blue). The shorter the PCA plot distance, the more similar the samples. (**B**) Cluster analysis of gene expression data. Differently colored squares represent the correlation between the two samples: red indicates higher correlation; blue indicates lower correlation. The legend is added in the top right corner. (**C**) Volcano plot of differentially expressed genes (DEGs) in *C. albicans*. Each dot represents a DEG. Significantly differentially expressed genes are shown as red dots (upregulated) or green dots (downregulated); others are indicated with grey dots. The abscissa represents fold change, and the ordinate represents statistical significance.

Principal component analysis (PCA) and cluster analysis were used to establish a visual representation of the correlation in transcriptomic data across different replicates (Figure 2A,B). PCA analysis demonstrated that the repeats for each sample were clustered together, while different groups were separated from each other at PC1 and PC2 levels. Based on sample correlation analysis, it was observed that the repeated samples of T_157 and CK_157 groups were highly correlated. Therefore, the difference between the groups was clear and the repeatability between samples was high. This ultimately demonstrated that the accuracy of the RNA-sequencing data used in the later analysis was reliable.

### 3.3. Total Analysis of DEGs

According to conventional threshold values of an adjusted *p*-value < 0.05 and |log_2_(FoldChange)| > 1, differentially expressed genes (DEGs) were uncovered in the transcriptome data of treatment groups (T_157 groups) compared with the control groups (CK_157). First, 554 genes exhibited differential expression after phosundoxin treatment, of which 420 genes were upregulated and 134 genes were downregulated (Figure 2C). The possible functions of the identified DEGs were predicted using the gene function classification system GO (Gene Ontology), which includes three ontologies, namely, molecular functions (MF), cellular components (CC), and biological processes (BP). The 554 annotated genes were categorized into 40 GO functional groups. Many of the genes were annotated in BP, followed by CC and MF, as shown in Table S3.

### 3.4. GO Enrichment Analysis of DEGs

**Figure 3:**
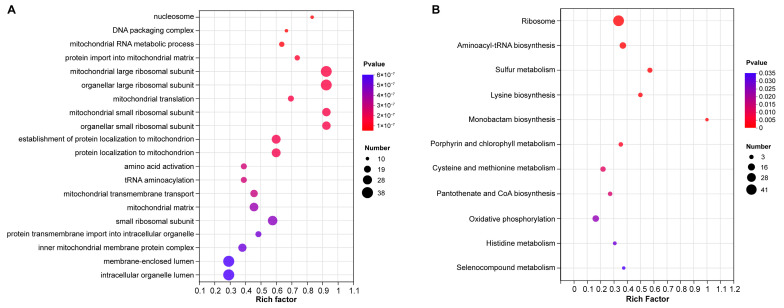
Functional enrichment analysis of DEGs. (**A**) GO enrichment analysis of DEGs based in the GO database. The top 20 terms of GO enrichment analysis in phosundoxin treatment and control groups. (**B**) KEGG enrichment analysis of DEGs based in KEGG database. The KEGG terms of KEGG enrichment analysis in phosundoxin treatment and control groups. The vertical axis represents the GO or KEGG term, and the horizontal axis represents the rich factor. Circle size reflects the number of genes involved in the pathway, and the color of the circle reflects its *p*-value (*p*-value < 0.05).

To fully appreciate the specific differences caused by phosundoxin, GO enrichment was used to analyze the DEGs of phosundoxin-treated *C. albicans* further. A total of 494 DEGs were classified into 319 significant terms, of which 197 were BP, 71 were CC, and 51 were MF (*p*-values < 0.05) (Table S4). The top 20 enriched terms can be found in Figure 3A. A high number of significant terms are related to mitochondria, including mitochondrial membrane, mitochondrial matrix, mitochondrial ribosomal subunits, mitochondrial respiratory chain complex assembly, mitochondrial transmembrane transport, and protein localization to mitochondria. These DEGs were also involved in metabolic and biosynthetic processes related to nucleotides including dTTP and ATP. In addition, some DEGs were related to the replication and translation of genetic material including DNA binding, DNA replication, DNA repair, structural constituents of ribosome, and tRNA aminoacylation for protein translation. Furthermore, DEGs involved in cell cycle processes including mitosis and meiosis were found.

### 3.5. KEGG Enrichment Analysis of DEGs

In order to uncover genes associated with the processed response, the DEGs were further enriched to terms in the KEGG database. A total of 136 DEGs were enriched in 11 significant pathways (*p*-values < 0.05) (Table S5, Figure 3B). Among the enriched pathways, many of the KEGG terms belong to genetic information processing, those involved in ribosome and aminoacyl-tRNA biosynthesis. Also, many belong to amino acid metabolism, including lysine, cysteine, methionine, histidine, and other amino acids. Furthermore, genes involved in oxidative phosphorylation (OXPHOS) belonging to energy metabolism were also enriched. Phosundoxin treatment impacted the respiratory chain complexes and specifically includes NADH: ubiquinone oxidoreductase, cytochrome-c reductase, cytochrome c oxidase, F_1_F_0_ ATP synthase (Table S5).

### 3.6. Effect of Phosundoxin Treatment on C. albicans

**Figure 4:**
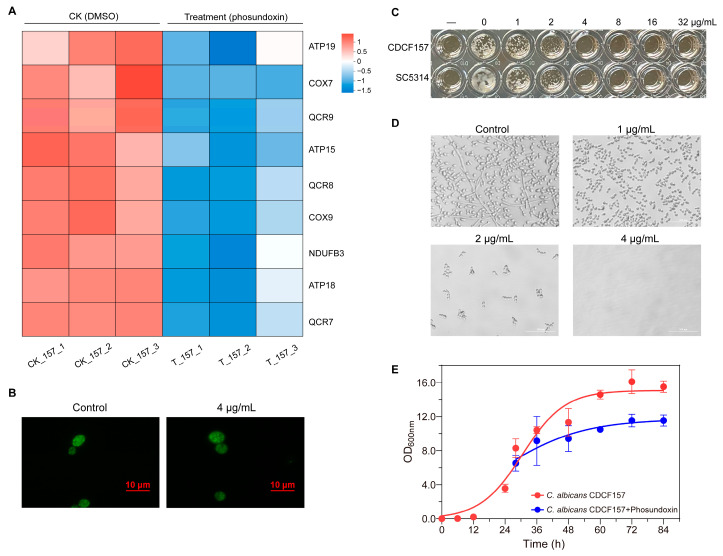
Effect of phosundoxin treatment on *C. albicans*. (**A**) Heatmap of differentially expressed genes (|log_2_(foldchange)| > 1, *p*-value < 0.05) involved in oxidative phosphorylation. Blue color indicates downregulated genes. (**B**) *C. albicans* CDCF157 treated with 4 mg/L phosundoxin was further incubated with mito-tracker green for visualizing mitochondrial architecture (×400). (**C**) The inhibitory effect of phosundoxin on the growth of azole-resistant *C. albicans* CDCF157 and azole-susceptible *C. albicans* SC5314. (**D**) Observation obtained from *C. albicans* CDCF157 treated with different concentrations of phosundoxin for 24 h (×400). (**E**) Growth condition of *C. albicans* CDCF157 in control groups and phosundoxin treatment.

According to the above results, genes related to OXPHOS, mitochondria, DNA replication, repair, translation, and the cell cycle were greatly impacted by exposure to phosundoxin. Among these, OXPHOS is the key process in the metabolism to generate ATP under aerobic conditions [24]. The genes involved in OXPHOS were selected and shown as a heatmap (Figure 4A).

Further analysis demonstrated that these genes were distributed in the oxidative respiratory chain, including NADH dehydrogenase (NDUFB3), cytochrome c reductase (QCR7, QCR8, QCR9), cytochrome c oxidase (COX7, COX9), and ATP synthase (ATP18, ATP19). Genes related to mitochondrial composition were mainly focused on mitochondrial ribosomal proteins, including 54S and 37S ribosomal proteins (e.g., YmL24, YmL37, MRPS16, YmL37, etc.). In addition, many of those associated with DNA replication repair and translation were mainly genes encoding DNA polymerase (POL30, DPB2, POL1), DNA replication protein (PSF1, PSF3) and tRNA ligases affecting amino acid metabolism (e.g., MSW1, MST1, MSM1). Genes impacting the cell cycle, including those involved in mitosis and meiosis, such as genes encoding cycle proteins (PCL2, CLB2), important transcriptional regulators (YOX1), and protein kinases in signal transduction (CDC5), were also found (Table 3).

Overall, phosundoxin may affect the function of mitochondria and disrupt OXPHOS process through entrance into mitochondria. We further examined if phosundoxin was localized to the mitochondria of *C. albicans* CDCF157 cells. Cellular localization in *C. albicans* was observed via fluorescence microscopy. As shown in Figure 4B, the mitochondrial architecture of *C. albicans* treated with phosundoxin became swollen and fragmented.

The inhibitory effects of 4 mg/L of phosundoxin on the growth of azole-resistant *C. albicans* CDCF157 and azole-susceptible *C. albicans* SC5314 were found in the antifungal susceptibility testing (Figure 4C,D). We verified the time–growth curve of *C. albicans* CDCF157 under phosundoxin treatment. Phosundoxin was added in the logarithmic phase of growth. It was observed that the growth of *C. albicans* CDCF157 was significantly reduced and gradually stabilized after 60 h, with a final cell increment of 66.7% of that without phosundoxin treatment (Figure 4E).

### 3.7. Verification of DEGs Using qRT-PCR

**Figure 5:**
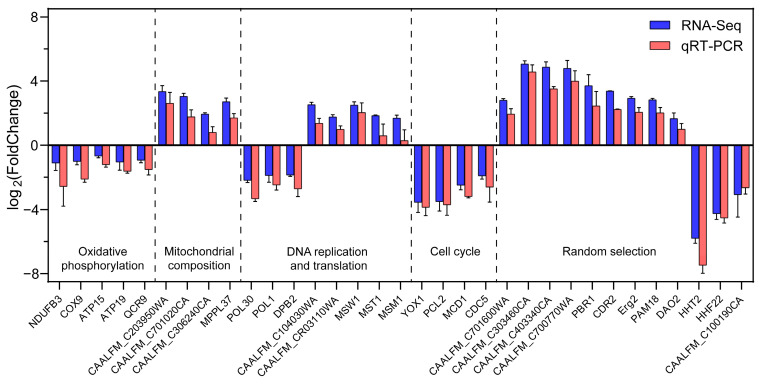
Expression levels of DEGs determined via transcriptome and qRT-PCR analyses after phosundoxin treatment. A total of 33 genes were selected for validation, of which 12 genes were randomly selected from 554 DEGs and 21 genes were associated with OXPHOS, mitochondria, replication, translation, and cell growth. Data were expressed as the means ± SE of three replicates.

To verify the above assumption, 12 DEGs, randomly selected, and 21 DEGs related to mitochondria, OXPHOS, cell growth, translation, replication, and repair in *C. albicans* were chosen and analyzed via qRT-PCR. As shown in Figure 5 and Table S6, the expression levels of the selected genes were consistent with transcriptomic data (R^2^ = 0.9795), suggesting that transcriptome data were highly reliable.

### 3.8. Acute Toxicity of Phosundoxin to Rats

Acute oral and percutaneous toxicity tests were performed with a dose of 5000 mg/kg for 14 days. The results showed that there were no dead and dying animals during the tests, which indicated that the LD_50_ of the phosundoxin is above 5000 mg/kg body weight in rats. All rats showed an increasing trend in body weight during the 14-day observation period and gross anatomy showed no abnormalities (Table 4). Furthermore, the clinical symptoms showed that no abnormalities were observed in the acute oral toxicity, while in the rats subjected to the acute percutaneous toxicity test, skin thickening appeared in the exposed area on the D3 and returned to normal within 10 days (Table S7). No other abnormalities were observed in clinical symptoms.

## 4. Discussion

Clinical use of some antifungal drugs is limited by toxicity and pathogenic fungal resistance developed by altering drug affinity and target abundance, reducing intracellular drug levels induced by efflux pumps [25,26]. It is important to explore novel, specific, and less-toxic drugs with new molecular targets for the treatment of fungal infections [6].

In this study, we demonstrated that phosundoxin, a novel TPP-conjugated compound, had a broad-spectrum of antifungal activity against many yeast-like and filamentous fungi, inhibiting the growth of most fungi in the concentration range from 2 to 4 mg/L (Table 1). *Trichophyton* is the leading cause of human skin and nail mycoses and has high worldwide prevalence [27,28,29]. Ketoconazole is commonly used for the treatment of infections caused by *Trichophyton* spp., of which the MIC has been reported to range from 16 to 128 mg/L [30]. In addition to ketoconazole, some traditional Chinese medicines, such as eugenol (MIC: 64–512 mg/L) and magnoflorine (MIC: 62.5 mg/L), have been able to -inhibit *Trichophyton*, but their MICs were relatively high [31]. In our study, phosundoxin inhibited *Trichophyton* growth at a level of 4–8 mg/L. Invasive aspergillosis remains a difficult-to-manage disease, especially when *Aspergillus* species with intrinsic or acquired resistance are involved [32,33,34]. All *Aspergillus* in this study exhibited the same MIC of 16 mg/L by phosundoxin. Invasive candidiasis may be caused by several *Candida* spp., including the common species *C. albicans*, as well as other pathogens, such as *C. tropicalis*, *C. parapsilosis*, and *C. krusei* [35]. *C. auris* is an emerging multidrug-resistant organism that poses a global threat [36]. The growth of these fungi was inhibited at a phosundoxin concentration of 4 mg/L. However, *C. glabrata* and its cryptic species had lower susceptibility to phosundoxin than other *Candida* species, with MICs from 8 to 16 mg/L, implying that the mitochondrial structure or regulation of *C. glabrata* and its cryptic species may differ from other *Candida* species [37]. In addition, we found that the MICs of the yeast phase were higher than that of hyphal phase when *Talaromyces marneffei* was exposed to phosundoxin, indicating that phosundoxin was more effective in inhibiting the hyphal phase of dimorphic fungi.

In particular, we found that phosundoxin had the same MICs (4 mg/L) for azole-resistant and azole-sensitive *C. albicans* isolates (Table 2). Clearly, the antifungal mechanism of phosundoxin, therefore, is different from that of azoles. Previously, it was shown that the lipophilicity of a TPP derivative governs its preferential localization to the mitochondria over the nucleus [12,14,38]. In this study, we found that phosundoxin could alter the expression of respiratory chain genes in *C. albicans*. The downstream metabolic processes of energy, amino acid, and nucleic acid were affected, ultimately leading to death of the fungi.

The oxidative respiratory chain in fungal mitochondria consists of four electron-transporting complexes (Ⅰ, Ⅱ, Ⅲ, and Ⅳ), controlling proton transportation and free energy release in OXPHOS. The released free energy is utilized by ATP synthase to synthesize ATP [39,40]. Gene *ndufb3* encodes an accessory subunit of the respiratory chain NADH dehydrogenase (complex Ⅰ), which is the first enzyme in the electron transport chain of mitochondria [40]. The restriction of *ndufb3* gene expression demonstrated that phosundoxin treatment undermined the assembly of NADH dehydrogenase, resulting in the hindrance of *C. albicans* OXPHOS at the beginning of the process (Table 3). QCR6, QCR7, QCR8, and QCR9, and QCR10 are involved in the assembly and mature process of cytochrome *bc1* complex Ⅲ [41]. Downregulation of the *qcr7*, *qcr8*, and *qcr9* genes by phosundoxin in this study may result in disruption of the functions of these three corresponding subunits, leading to an overall decrease in cytochrome b-c1 complex synthesis (Table 3). In addition, cytochrome c oxidase (complex Ⅳ) is a terminal enzyme of the electron transport chain. In particular, *cox7* and *cox9*, which encode the cytochrome c oxidase subunit Ⅶa, are essential for stable assembly of the functional enzymes. Deletion mutations in *cox7* or *cox9* genes resulted in loss of detectable cytochrome c oxidase, as well as of activity in yeast [42,43]. Downregulation of the *cox7* and *cox9* genes by phosundoxin in this study may result in interruption of the mentioned subunits functions and further inhibit the ability of cytochrome c oxidase to transfer electrons from reduced cytochrome c to the mitochondrial intermembrane space of *C. albicans* (Table 3).

F_1_F_0_-ATP synthase is responsible for the synthesis of more than 90% of intracellular ATP [44]. *atp18* and *atp19*, encoding i and k subunits in the F_1_F_0_-ATP synthase, respectively, are involved in the stepwise assembly of F_1_F_0_-ATP synthase dimers and oligomers [45]. The downregulation of *atp18* and *atp19* genes (Table 3) resulted in a reduction in the interaction between F_1_ and F_0_, subsequently altering the stability of F_1_F_0_-ATP synthase. F_1_F_0_-ATP synthase has a relatively conserved overall structure from fungi to mammals. However, the i and k subunits were specific to the fungal F_1_F_0_-ATP synthase, which implies that the i and k subunits may be new targets of antifungal drugs [45].

**Figure 6:**
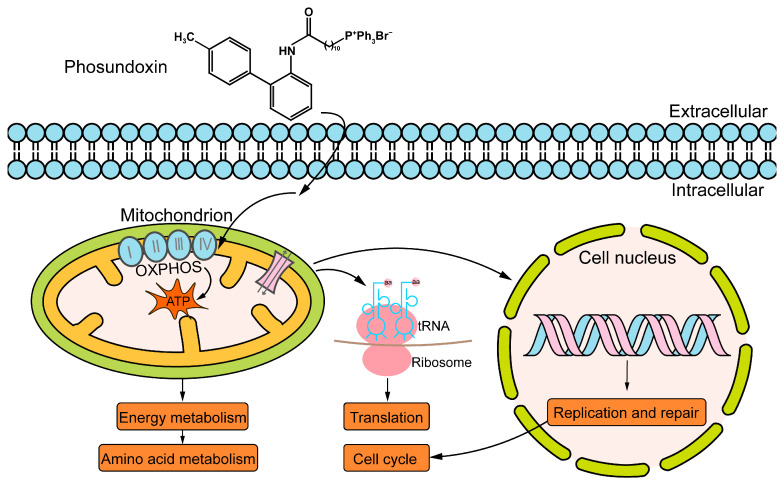
A schematic diagram illustrating antifungal effects of phosundoxin on *C. albicans*.

Phosundoxin was synthesized based on TPP^+^-conjugated boscalid analogues. Boscalid had effective antifungal activity as the mitochondrial respiratory chain inhibitor [46]. However, resistance to boscalid has increased rapidly [47,48]. In our study, phosundoxin had a broad-spectrum of antifungal activity against common fungal pathogens and mainly inhibited OXPHOS complexes and ATP synthase. Overall, the mechanism of phosundoxin inhibiting *C. albicans* begins as it passes through the cell membrane, enters the mitochondria, destroys mitochondrial structure, and thereafter interferes with the electron transfer and ATP generation in the mitochondria. Finally, it impacts amino acid metabolism and the processing of genetic information, including the replication and translation processes, and ultimately abolishes mitosis and destroys the cell cycle, thereby inhibiting the growth of *C. albicans* (Figure 6). However, further studies are needed to determine the key mechanisms that *C. albicans* responses to phosundoxin. The acute toxicity results of phosundoxin were mild toxicity (Table 4), but the clinical safety and efficacy need to be further studied to confirm the development of this compound as a topical or oral drug. In addition, the effect of phosundoxin on *C. glabrata*, *C. parapsilosis*, and *C. auris*, as well as *A. fumigatus*, is also something which will gradually be studied.

## Figures and Tables

**Table 1:** Antifungal susceptibilities of phosundoxin compound for *Candida* spp., *Cryptococcus* spp., *Aspergillus* spp., *Trichophyton* spp., and *Talaromyces* spp. isolates.

Genus/Species (No. of Isolates)	Range (mg/L)	MIC_50_^a^ (mg/L)	MIC_90_^b^ (mg/L)	MIC/GM MIC ^c^ (mg/L)
*Candida* (130)				
*C. albicans* (18)	4	4	4	4
*C. tropicalis* (20)	2–4	2	4	2.46
*C. krusei* (11)	4	4	4	4
*C. auris* (3)	4	-	-	4
*C. parapsilosis* (11)	2–4	4	4	3.76
*C. metapsilosis* (10)	2–4	4	4	3.25
*C. orthopsilosis* (10)	4	4	4	4
*C. haemulonii* (10)	4	4	4	4
*C. haemulonii var. vulnera* (4)	4	-	-	4
*C. duobushaemulonii* (10)	2–4	4	4	3.73
*C. glabrata* (10)	8–16	16	16	13.93
*C. nivariensis* (10)	8	8	8	8
*C. bracarensis* (3)	8	-	-	8
*Cryptococcus* (10)				
*C. neoformans* (10)	4	4	4	4
*Aspergillus* (4)				
*A. niger* (1)	-	-	-	16
*A. terreus* (1)	-	-	-	16
*A. nidulans* (1)	-	-	-	16
*A. fumigatus* (1)	-	-	-	16
*Trichophyton* (6)				
*T. rubrum* (3)	8	-	-	8
*T. mentagrophytes* (2)	8	-	-	8
*T. soudanense* (1)	-	-	-	4
*Talaromyces* (8)				
*T. marneffei* (Filamentous) (8)	2–8	4	4	3.67
*T. marneffei* (Yeast-like) (8)	2–8	8	8	5.66

MICs of phosundoxin determined by the CLSI M27 and M38 reference method that resulted in 100% inhibition of growth, compared with growth control. ^a^ MIC_50_, MIC at which 50% of test isolates were inhibited. ^b^ MIC_90_, MIC at which 90% of test isolates were inhibited. ^c^ Geometric mean MICs. The MIC_50_, MIC_90_, and GM MIC values were ascertained for species where at least eight isolates, respectively, were tested. Range indicates MIC range, that is, the range from the minimum to maximum MIC value in the tested strains.

**Table 2:** Antifungal susceptibilities of four compounds for 10 azole-susceptible *C. albicans* and 8 azole-nonsusceptible *C. albicans* isolates.

Genus/Species (No. of Isolates)	Antifungal	Range	MIC_50_	MIC_90_	MIC/GM MIC
azole-susceptible *C. albicans* (10)	Phosundoxin	4	4	4	4
	FLC	0.25–4	0.25	2	0.73
	ITC	0.015–0.25	0.06	0.25	0.09
	VRC	<0.008–0.25	<0.008	0.12	-
azole-nonsusceptible *C. albicans* (8)	Phosundoxin	4	4	4	4
	FLC	4–32	16	32	16
	ITC	0.25–1	1	1	0.71
	VRC	0.5–4	1	4	1.09

Abbreviations: MIC, minimum inhibitory concentration; ITC, itraconazole; VRC, voriconazole; FLC, fluconazole. MICs of phosundoxin are determined via the CLSI M27-A4 reference method. MICs of the remaining antifungal agents determined using the Sensititre^®^ YeastOne^®^ YO10 panel as described in the Methods. The MIC_50_, MIC_90_, and GM MIC values were ascertained for species where at least eight isolates, respectively, were tested.

**Table 3:** Impacted genes after phosundoxin treatment related to oxidative phosphorylation (OXPHOS), mitochondria, DNA replication, repair, translation, and cell cycle.

Category	Name	Gene Description	FC	Log_2_FC
Oxidative phosphorylation	Ndufb3	NADH: ubiquinone oxidoreductase subunit B3	0.38	−1.41
	QCR9	cytochrome-c reductase subunit 9	0.40	−1.32
	QCR8	cytochrome-c reductase subunit 8	0.48	−1.06
	QCR7	cytochrome-c reductase subunit 7	0.47	−1.10
	COX7	cytochrome c oxidase subunit VIIa	0.43	−1.23
	COX9	cytochrome c oxidase subunit VIIa	0.38	−1.39
	ATP19	F_1_F_0_ ATP synthase subunit k	0.38	−1.39
	ATP18	F_1_F_0_ ATP synthase subunit i	0.48	−1.05
	ATP15	F_1_F_0_ ATP synthase subunit ε	0.49	−1.04
Mitochondrial composition	CAALFM_C203950WA	mitochondrial 54S ribosomal protein YmL24/14	11.01	3.46
	MRPL37	mitochondrial 54S ribosomal protein YmL37	6.32	2.66
	CAALFM_C306240CA	mitochondrial 54S ribosomal protein YmL39	3.07	1.62
	CAALFM_C701020CA	mitochondrial 37S ribosomal protein MRPS16	8.42	3.07
	CAALFM_C108920WA	mitochondrial 37S ribosomal protein RSM25	3.49	1.80
DNA replication repair and translation	POL30	proliferating cell nuclear antigen	0.23	−2.13
	POL1	DNA-directed DNA polymerase α catalytic subunit	0.30	−1.74
	DPB2	DNA polymerase epsilon noncatalytic subunit	0.29	−1.77
	PSF3	DNA replication protein	0.43	−1.21
	PSF1	DNA replication protein	0.46	−1.11
	CK2B	90S pre-ribosome components UTP-C complex	0.03	−4.86
	MSM1	Methionine-tRNA ligase	3.48	1.80
	MST1	Threonine-tRNA ligase	3.82	1.93
	MSW1	Tryptophan-tRNA ligase	6.09	2.61
Cell cycle	PCL2	cyclin	0.09	−3.41
	CLB2	β-type cyclin	0.40	−1.33
	YOX1	Yox1p	0.09	−3.42
	CDC5	polo kinase	0.29	−1.80

**Table 4:** Acute oral and percutaneous toxicity results of the compound phosundoxin.

Observation	Acute Oral Toxicity	Acute Percutaneous Toxicity
Deaths	N	N
Weight	Normal growth	Normal growth
Anatomical result	N	N
Clinical observation ^a^	N	D3: Skin thickening at the site of drug contact;D10: Completely back to normal

^a^ Clinical observations include salivation, diarrhea, tremors, convulsions, eye lacrimation, respiration problem, motor activity, diarrhea, skin problems. N: no abnormalities.

## Data Availability

Data are contained within the article and Supplementary Materials.

## Supplementary Materials

The following supporting information can be downloaded at https://www.mdpi.com/article/10.3390/jof10010028/s1: Table S1: Statistical table of original data obtained by sequencing and data after quality control; Table S2: Results of comparison between the original data (clean reads) and the reference genome after quality control; Table S3: Annotation results of DEGs in the GO database; Table S4: The GO enrichment analysis of DEGs in the GO database; Table S5: The KEGG enrichment analysis of DEGs in the KEGG database; Table S6: Primers used for RT-qPCR; Table S7: Clinical symptoms of acute percutaneous toxicity test.
