# Supplementary material for: A Novel Mitochondrial Targeted Compound Phosundoxin Showing Potent Antifungal Activity against Common Clinical Pathogenic Fungi
Source: J Fungi (Basel). 2023 Dec 31;10(1):28. doi: 10.3390/jof10010028 (PMC10817537; doi:10.3390/jof10010028)
Supplement: Supplementary file 1 [file jof-10-00028-s001.zip › Supplementary Materials/Table S1.docx]

**Table S1. Statistical table of original data obtained by sequencing and data after quality control.**

| **Sample** | **Raw reads** | **Raw bases** | **Clean reads** | **Clean bases** | **Error rate (%)** | **Q20 (%)** | **Q30 (%)** | **GC content (%)** |
| --- | --- | --- | --- | --- | --- | --- | --- | --- |
| **T157_3** | 48381410 | 7305592910 | 47049182 | 6960046974 | 0.0255 | 97.84 | 93.79 | 37.76 |
| **T157_2** | 52133122 | 7872101422 | 51015118 | 7517445878 | 0.0255 | 97.85 | 93.78 | 37.64 |
| **T157_1** | 57236446 | 8642703346 | 55753238 | 8212131385 | 0.0252 | 97.95 | 94.03 | 37.53 |
| **CK157_3** | 48200324 | 7278248924 | 46972832 | 6820744698 | 0.0251 | 98.01 | 94.2 | 37.75 |
| **CK157_2** | 44026426 | 6647990326 | 42654490 | 6260499269 | 0.0251 | 97.98 | 94.23 | 37.83 |
| **CK157_1** | 58268584 | 8798556184 | 57134444 | 8352008282 | 0.0251 | 98.01 | 94.15 | 37.94 |
