# Supplementary material for: A Novel Mitochondrial Targeted Compound Phosundoxin Showing Potent Antifungal Activity against Common Clinical Pathogenic Fungi
Source: J Fungi (Basel). 2023 Dec 31;10(1):28. doi: 10.3390/jof10010028 (PMC10817537; doi:10.3390/jof10010028)
Supplement: Supplementary file 1 [file jof-10-00028-s001.zip › Supplementary Materials/Table S2.docx]

**Table S2. Results of comparison between the original data (clean reads) and the reference genome after quality control.**

| **Sample** | **Total reads** | **Total mapped** | **Multiple mapped** | **Uniquely mapped** |
| --- | --- | --- | --- | --- |
| **T157_3** | 47049182 | 44448626(94.47%) | 1019777(2.17%) | 43428849(92.31%) |
| **T157_2** | 51015118 | 48137315(94.36%) | 1080799(2.12%) | 47056516(92.24%) |
| **T157_1** | 55753238 | 52712848(94.55%) | 1190651(2.14%) | 51522197(92.41%) |
| **CK157_3** | 46972832 | 44418897(94.56%) | 1021142(2.17%) | 43397755(92.39%) |
| **CK157_2** | 42654490 | 40355596(94.61%) | 935864(2.19%) | 39419732(92.42%) |
| **CK157_1** | 57134444 | 54000479(94.51%) | 1313020(2.3%) | 52687459(92.22%) |
