# Supplementary material for: A Novel Mitochondrial Targeted Compound Phosundoxin Showing Potent Antifungal Activity against Common Clinical Pathogenic Fungi
Source: J Fungi (Basel). 2023 Dec 31;10(1):28. doi: 10.3390/jof10010028 (PMC10817537; doi:10.3390/jof10010028)
Supplement: Supplementary file 1 [file jof-10-00028-s001.zip › Supplementary Materials/Table S6.docx]

**Table S6.** Primers used for RT-qPCR

| **Gene name** | **Gene ID** | **Primer forward (5′-3′) /Primer reverse (5′-3′)** |
| --- | --- | --- |
| ACT1 | CAALFM_C113700WA | ACCGAAGCTCCAATGAATCCAAAATCC |
|  |  | GTTTGGTCAATACCAGCAGCTTCCAAA |
| NDUFB3 | CAALFM_C108930CA | TACCAGTAGTAATGGCAATC |
|  |  | TATACCTAATCCAGGGAATG |
| COX9 | CAALFM_C205930WA | CTCTTGCTCCAATCACAG |
|  |  | ATGTTCGACTTCCCAGTA |
| ATP15 | CAALFM_C603130WA | ACAAACAAGCTGGTGTATCA |
|  |  | TTGGCTTCAACAAATCCTCT |
| ATP19 | CAALFM_C112320CA | ATGGGTGCTGCTTATCA |
|  |  | GGTTGGACTTGGTGGAC |
| QCR9 | CAALFM_C402890CA | CAATCTACGTTGCCACTATCTTTG |
|  |  | TGTTCCTCCCACCATTTGTTC |
| CAALFM_C203950WA | CAALFM_C203950WA | CCCCGTATATCGTGTGTGTCA |
|  |  | GTTGTCTGGACGTTCTTGGC |
| CAALFM_C701020CA | CAALFM_C701020CA | TATCAGGTAATGACTCGGGTTTG |
|  |  | CCATGTAACACGAGGAGTAGGAA |
| CAALFM_C306240CA | CAALFM_C306240CA | AAAGCTAGAGCAAATACCACCA |
|  |  | GGCAATGTCTCTTAGCTCTTGG |
| MRPL37 | CAALFM_C104880CA | ATCATGCCCTGCCGGTACT |
|  |  | AAGCATGGTCCATAACCATTCAG |
| POL30 | CAALFM_C401770WA | TAGTTTAGATGACCCAGTT |
|  |  | CAAACAATGCAGGAGTT |
| POL1 | CAALFM_C304300CA | GCGTTGTATTATGCTGGATGG |
|  |  | AAACAGAAATCTGCCTTGTCGT |
| DPB2 | CAALFM_CR09900CA | ACTATTTATTGATGGCGATGGG |
|  |  | CTAGGGTGTCAGTTCGATGAGC |
| CAALFM_C104030WA | CAALFM_C104030WA | TCTGGGCAGACTTCTCATGTTAC |
|  |  | TCACCACGTACTTTCAAAGGATC |
| CAALFM_CR03110WA | CAALFM_CR03110WA | GGTGAAAGTACCCAATGACCAA |
|  |  | TGAGGGATGGCTAAACCTGAT |
| MSW1 | CAALFM_C502770WA | TGCAGCCATTGCCAGTTTA |
|  |  | AGTTGTTGTGCCTTCAGTTTCC |
| MST1 | CAALFM_C102930CA | GGCGAAGGAAATTGGGCTAC |
|  |  | CTGAATGGTCCCTACTTGGTGT |
| MSM1 | CAALFM_C504650CA | TCAAGCCAACCAAATCTTCCA |
|  |  | CTCGTGGTCTCAGCACATAAGTAA |
| PCL2 | CAALFM_C108570CA | TACGCAATCTATTACCTGCCAATG |
|  |  | GATTTAGCAGCAACTATCAAAGCAC |
| YOX1 | CAALFM_C700970CA | CATCCACCAGTTCATCAGTTGC |
|  |  | CCTTGTTGTCAGTAGGAGTAGAGGC |
| MCD1 | CAALFM_CR10540CA | TTGCCTTCAAACTCAGAACACC |
|  |  | CCAAACCTTCCATTGCCTCT |
| CDC5 | CAALFM_C100950CA | CGGATAAGACATACGAAGTCAGTTT |
|  |  | TAGGTGATAGCGACAGAGGAAGTA |
| CAALFM_C701600WA | CAALFM_C701600WA | TTCCTGCCGTCCAATTACCA |
|  |  | TGACCAGTTTCACCACCCAC |
| CAALFM_C303460CA | CAALFM_C303460CA | ACAACAGCAGCAATGACACA |
|  |  | GTTGGAGTTGGTGGTGGAGA |
| CAALFM_C403340CA | CAALFM_C403340CA | TTATTGGCGGTGGTGCTGTT |
|  |  | AGGATCGGCTCTGGTGGTAA |
| CAALFM_C700770WA | CAALFM_C700770WA | TGCCACCACAGCTACATCTAC |
|  |  | GGTGTTGCCGTGGGTGTTAT |
| PBR1 | CAALFM_C106370CA | TGCTGCTGGTTCTGATGGTAT |
|  |  | CTTTGGTGGCAGATTTGGATT |
| CDR2 | CAALFM_C304890WA | GCTGGTCAAATCACTTC |
|  |  | ACTGAGTCTGTCGGTTC |
| ERG2 | CAALFM_C100800CA | CCTGGTGCATTGATTCCCGA |
|  |  | TGATTCACCGGGCATAGCAT |
| PAM18 | CAALFM_C400520WA | TGGCACCATTAGAAGCACCAA |
|  |  | TCCTTCGGCAGCTTTCTTCT |
| DAO2 | CAALFM_C403380CA | GCTGGAGCTGGTATTCTCGG |
|  |  | GAAAATGAGCACCAGCCCAC |
| HHT2 | CAALFM_CR06810WA | TTCCAAAGATTAGTCAGA |
|  |  | GAATAAACCAACCAAGTA |
| HHF22 | CAALFM_CR06800CA | GCTTTGATTTATGAAGAAG |
|  |  | TCCAATGAAGTGACGGT |
| CAALFM_C100190CA | CAALFM_C100190CA | CTGTGACTCGTGCTTGACAC |
|  |  | ACTTCGGTTTCTTCTTCAGGGA |
