# Supplementary material for: A Novel Mitochondrial Targeted Compound Phosundoxin Showing Potent Antifungal Activity against Common Clinical Pathogenic Fungi
Source: J Fungi (Basel). 2023 Dec 31;10(1):28. doi: 10.3390/jof10010028 (PMC10817537; doi:10.3390/jof10010028)
Supplement: Supplementary file 1 [file jof-10-00028-s001.zip › Supplementary Materials/Table S7.docx]

**Table S7.** Clinical symptoms of acute percutaneous toxicity test

| **Dose**  **(mg/kg)** | **Sex** | **No.** | **Clinical symptoms** | | | | | | | | | | | | | | |
| --- | --- | --- | --- | --- | --- | --- | --- | --- | --- | --- | --- | --- | --- | --- | --- | --- | --- |
|  |  |  | **D1** | **D2** | **D3** | **D4** | **D5** | **D6** | **D7** | **D8** | **D9** | **D10** | **D11** | **D12** | **D13** | **D14** | **D15** |
| 5000 | ♀ | 1F01 | N | N | C21 | C21 | C21 | C21 | C21 | C21 | C21 | C21NP | N | N | N | N | N |
|  |  | 1F02 | N | N | C21 | C21 | C21 | C21 | C21 | C21 | C21 | C21NP | N | N | N | N | N |
|  |  | 1F03 | N | N | C21 | C21 | C21 | C21 | C21 | C21 | C21 | C21NP | N | N | N | N | N |
|  |  | 1F04 | N | N | C21 | C21 | C21 | C21 | C21 | C21NP | N | N | N | N | N | N | N |
|  |  | 1F05 | N | N | C21 | C21 | C21 | C21 | C21 | C21NP | N | N | N | N | N | N | N |
| 5000 | ♂ | 1M01 | N | N | C21 | C21 | C21 | C21 | C21NP | N | N | N | N | N | N | N | N |
|  |  | 1M02 | N | N | C21 | C21 | C21 | C21 | C21 | C21NP | N | N | N | N | N | N | N |
|  |  | 1M03 | N | N | C21 | C21 | C21 | C21 | C21 | C21 | C21 | C21NP | N | N | N | N | N |
|  |  | 1M04 | N | N | C21 | C21 | C21 | C21 | C21 | C21NP | N | N | N | N | N | N | N |
|  |  | 1M05 | N | N | C21 | C21 | C21NP | N | N | N | N | N | N | N | N | N | N |

N: No abnormalities.

C21: Skin thickening appeared in the compound 20201 exposed area.

NP: The symptom no longer exists.
